# Non-steroidal anti-inflammatory drug hypersensitivity: association with elevated basal serum tryptase?

**DOI:** 10.1186/1710-1492-10-19

**Published:** 2014-04-24

**Authors:** Cornelia S Seitz, Knut Brockow, Johannes Hain, Axel Trautmann

**Affiliations:** 1 Department of Dermatology and Allergy, University Hospital Göttingen, Göttingen, Germany; 2 Department of Dermatology and Allergy, Biederstein, Technische Universität München, Munich, Germany; 3 Institute of Mathematics, Department of Statistics, University of Würzburg, Würzburg, Germany; 4 Department of Dermatology and Allergy, University Hospital Würzburg, Josef Schneider Strasse 2, Würzburg 97080, Germany

**Keywords:** Anaphylaxis, Non-steroidal anti-inflammatory drug, Mastocytosis, Drug allergy, Drug reaction, Pseudo-allergy

## Abstract

**Background:**

It is hypothesized that because of higher mast cell numbers and mediator release, mastocytosis predisposes patients for systemic immediate-type hypersensitivity reactions to certain drugs including non-steroidal anti-inflammatory drugs (NSAID).

**Objective:**

To clarify whether patients with NSAID hypersensitivity show increased basal serum tryptase levels as sign for underlying mast cell disease.

**Methods:**

As part of our allergy work-up, basal serum tryptase levels were determined in all patients with a diagnosis of NSAID hypersensitivity and the severity of the reaction was graded. Patients with confirmed IgE-mediated hymenoptera venom allergy served as a comparison group.

**Results:**

Out of 284 patients with NSAID hypersensitivity, 26 were identified with basal serum tryptase > 10.0 ng/mL (9.2%). In contrast, significantly (*P* = .004) more hymenoptera venom allergic patients had elevated tryptase > 10.0 ng/mL (83 out of 484; 17.1%). Basal tryptase > 20.0 ng/mL was indicative for severe anaphylaxis only in venom allergic subjects (29 patients; 4x grade 2 and 25x grade 3 anaphylaxis), but not in NSAID hypersensitive patients (6 patients; 4x grade 1, 2x grade 2).

**Conclusions:**

In contrast to hymenoptera venom allergy, NSAID hypersensitivity do not seem to be associated with elevated basal serum tryptase levels and levels > 20 ng/mL were not related to increased severity of the clinical reaction. This suggests that mastocytosis patients may be treated with NSAID without special precautions.

## Background

Anaphylaxis is defined as a systemic hypersensitivity reaction, which can be sub-classified as allergic anaphylaxis when the reaction is mediated by an immune mechanism, or non-allergic anaphylaxis induced by non-immunologic triggers (formerly called anaphylactoid reaction) [1]. Non-steroidal anti-inflammatory drugs (NSAID) are the most common causes of non-allergic drug-induced systemic hypersensitivity reactions [2]. In the pathogenesis of non-allergic anaphylactic reactions, mast cell activation, complement system activation, inhibition of cyclooxygenase 1, up-regulation of endothelial cell-active mediators, and specific enzyme defects are discussed [3,4].

Mastocytosis comprises a heterogeneous disease spectrum with increased mast cell burden mainly in skin and bone marrow. Less frequently, involvement of the gastrointestinal tract, lymph nodes, spleen, and liver occur. Anaphylaxis symptoms may develop due to liberation of mast cell mediators whereas chronic organ dysfunction is caused by progressive tissue infiltration with mast cells. Known non-immunologic trigger factors for liberation of mast cell mediators are specific physical stimuli (such as skin rubbing, heat or physical exercise) and certain drugs [5]. While some observations suggest a possible increase of risk and severity for non-allergic systemic hypersensitivity induced by NSAID in mastocytosis patients, confirmatory data is sparse [6]. Nevertheless, NSAID are still considered as triggering anaphylactic reactions in patients with mast cell disease, probably because this group of compounds accounts for most non-allergic hypersensitivity reactions overall.

Aim of this retrospective analysis was to evaluate the relevance of basal serum tryptase measurement in patients with NSAID hypersensitivity reactions using a cohort of clinically and diagnostically well-defined patients. Basal serum tryptase values and grade of anaphylaxis were compared with a cohort of patients suffering from hymenoptera venom allergy.

## Methods

### Patients with NSAID hypersensitivity

From 2004 to 2011, all patients referred to the allergy clinic of Würzburg with a history of hypersensitivity reactions induced by NSAID were subjected to standardized allergy diagnostics including determination of basal serum tryptase. Written informed consent for allergy work-up was obtained and the local Ethics Committee of the Faculty of Medicine, University of Würzburg, Germany approved data collection and evaluation.

### Allergy testing

We performed prick and intradermal tests on the volar forearm with a series of NSAID, including the culprit drug as described previously [7-9]. Test procedures and readings after 15 minutes were performed according to the EAACI recommendations [10]. In individual cases allergic or non-allergic hypersensitivity to other concomitantly administered drugs and/or other exposed antigens (e.g. natural rubber latex from stoppers, tubes or gloves) which may have been responsible for the symptoms as well, were excluded by skin and provocation testing. All agents were freshly reconstituted, and physiological saline solution was used as negative control. Provocation testing with NSAID was done according to published protocols using standardized doses [7]. General principles of our protocol were as follows: (a) the time interval since the hypersensitivity reaction was at least 2 weeks; (b) during the entire provocation procedure the patient was observed and equipment for emergency treatment was available; (c) the dosage increased stepwise to a normal dose with intervals of 1hour between the individual doses; (d) strict adherence to absolute and relative contraindications for drug challenge tests; (e) prior to provocation testing written informed consent was obtained from each patient.

### Hymenoptera venom allergic patients

During 2004 to 2011, basal serum tryptase levels of patients with anaphylactic IgE-mediated reactions after bee/wasp stings (diagnosis by positive serum-IgE and/or skin testing) eligible for venom immunotherapy were determined. This cohort of clinically and diagnostically well-defined patients served as a comparison group.

### Grading of anaphylaxis

The reported hypersensitivity symptoms were classified according to severity (Table 1) [11]. Grade 1 consists of cutaneous symptoms such as urticaria and angioedema; grade 2 (moderate) and 3 (severe anaphylaxis) include symptoms of the following organ systems: cardiovascular (hypotension, tachycardia), respiratory (dyspnoea, bronchoconstriction), and digestive tract (vomiting, abdominal pain, incontinence). Only patients with clearly documented objective symptoms of at least grade 1 anaphylaxis were included.

**Table 1:** **Grading system for anaphylaxis (modified from**[11]**)**

**Grade**	**Symptoms**
1 (mild): skin and subcutaneous tissue only	Generalized urticaria and/or angioedema (e.g. periorbital oedema, lip oedema)
2 (moderate): features suggesting respiratory, cardiovascular and/or gastrointestinal involvement	Dyspnoea, chest or throat tightness, stridor, wheeze, nausea, abdominal pain, vomiting, tachycardia, dizziness (presyncope)
3 (severe): hypoxia, hypotension and neurological compromise	Cyanosis, hypotension (systolic blood pressure < 90 mm Hg in adults), confusion, collapse, loss of consciousness, loss of sphincter control (incontinence)

### Tryptase measurement

For determination of baseline serum tryptase levels (obtained at least 1 week after resolution of the clinical signs) commercially available ImmunoCAP™ Tryptase was used, an *in-vitro* test for the quantitative measurement of both alpha and beta tryptase concentration in human serum [12].

### Diagnosis of mastocytosis

Criteria for classification and diagnosis of mastocytosis were applied as published in recent consensus papers [13,14].

### Statistical analysis

To compare the group of hymenoptera venom allergic patients with the group suffering from NSAID hypersensitivity, the Mann–Whitney test was conducted in case of a metrically scaled outcome variable. For nominal outcome variables we used either the Chi-square test or Fisher’s exact test depending on the number of outcome categories. All tests were two-tailed and a *P* value smaller than .05 was considered statistically significant. The analysis was done using SPSS version 21 for Windows (SPSS Inc., Chicago, IL).

## Results

### Patients with NSAID hypersensitivity

284 patients, 203 females and 81 males, presenting with hypersensitivity symptoms induced by NSAID, were evaluated (Table 2). The average age at the time of the reaction was 51 years (ranging from 10 to 89 years). NSAID were administered orally (n = 249), intravenously (n = 14), intramuscularly (n = 19) or in form of a suppository (n = 2). 256 patients had experienced NSAID hypersensitivity within 12 months prior to testing, 22 between 1 and 5 years, and 6 between 6 and 10 years prior to testing.

**Table 2:** Clinical data and tryptase values of patients with NSAID hypersensitivity as well as the hymenoptera venom allergy group

	**NSAID**	**Comparison group: hymenoptera venom allergy**	***P *** **values**
Number of patients	284	484	n.a.
Mean age (range)	51 (10–89)	47 (7–81)	.026
Male/female	81/203	287/197	< .001
Severity of anaphylaxis			< .001
Grade 1	174 (61.3%)	26 (5.4%)	
Grade 2	110 (38.7%)	249 (51.4%)	
Grade 3	0 (0.0%)	209 (43.2%)	
Application			n.a.
Oral	249	n.a.	
Intravenous	14	n.a.	
Intramuscular	19	n.a.	
Suppository	2	n.a.	
Basal serum tryptase			.004
Minimum [ng/mL]	0.0	0.0	
Maximum [ng/mL]	94.8	148.0	
Mean [ng/mL]	5.9	7.8	
0.0 to 5.0 ng/mL	166 (58.5%)	276 (57.0%)	
> 5.0 to 10.0 ng/mL	92 (32.4%)	125 (25.8%)	
> 10.0 ng/mL	26 (9.2%)	83 (17.1%)	
> 10.0 to 20.0 ng/mL	20 (7.0%)	54 (11.2%)	
> 20.0 to 50.0 ng/mL	5 (1.8%)	24 (5.0%)	
> 50.0 ng/mL	1 (0.4%)	5 (1.0%)	

*P* values calculated from the comparison between patients with NSAID hypersensitivity and hymenoptera venom allergy. n.a., not applicable.

### Allergy testing

Definite confirmation of non-allergic NSAID hypersensitivity was achieved by positive provocation testing in all 284 patients with negative NSAID skin tests. The type of clinical NSAID hypersensitivity was urticaria/angioedema in all patients; in 201 exacerbation of the symptoms occurred in subjects with underlying chronic spontaneous urticaria and in the remaining 83 of patients urticaria/angioedema developed without an obvious history of chronic urticaria. Therefore, 70.8% of patients could be classified as NSAID-exacerbated cutaneous disease (NECD) and 29.2% as NSAID-induced urticaria/angioedema (NIUA) as proposed by a recent position paper [15]. Besides urticaria/angioedema, in 110 patients (38.7%, Table 2) symptoms suggesting respiratory, cardiovascular or gastrointestinal involvement such as dyspnoea, throat tightness, nausea, abdominal pain, tachycardia or dizziness developed during NSAID provocation testing.

### Hymenoptera venom allergic patients

Association of hymenoptera venom allergy with elevated basal serum tryptase has recently been shown in several studies [5,16-18]. Accordingly, of the 484 patients in the comparison group suffering from hymenoptera venom allergy, a significant number with elevated tryptase levels and severe anaphylactic reactions were identified (Table 2).

### Tryptase measurement

Details of the 6 NSAID hypersensitive patients with basal serum tryptase levels > 20 ng/mL are shown in Table 3. Four of these suffered from urticaria/angioedema whereas 2 patients experienced moderate (grade 2) anaphylaxis. This was in contrast to the 29 hymenoptera venom allergic patients with tryptase levels > 20 ng/mL, where 25 developed severe (grade 3) anaphylaxis (Table 4).

**Table 3:** **Characteristics of patients with NSAID hypersensitivity and basal serum tryptase level > 20** n**g/mL**

**Patient**	**Age, sex**	**Drug**	**Application**	**Latency [minutes]**	**Anaphylaxis [grade]**	**Treatment**	**Tryptase [ng/mL]**	**Classification of Mastocytosis**
#1	54, f	Acetylsalicylic acid	Oral	15	1	H_1_-blocker	39.7	ISM
#2	61, f	Acetylsalicylic acid	Oral	30	1	No	42.2	ISM
#3	72, f	Metamizol	Oral	20	1	No	23.5	CM
#4	54, f	Diclofenac	Oral	20	1	No	32.7	n.d.
#5	37, f	Acetylsalicylic acid	Oral	30	2	H_1_-blocker, steroids	26.2	n.d.
#6	71, f	Diclofenac	Oral	10	2	H_1_-blocker	94.8	ISM

ISM, indolent systemic mastocytosis. CM, cutaneous mastocytosis. n.d., not determined yet.

**Table 4:** **Characteristics of patients with hymenoptera venom allergy and basal serum tryptase level > 20** n**g/mL**

**Patient**	**Age, sex**	**Stinging insect**	**Anaphylaxis [grade]**	**Tryptase [ng/mL]**	**Classification of Mastocytosis**
#1	54, m	Wasp	3	27.2	n.d.
#2	60, m	Wasp	3	88.3	ISM
#3	65, f	Wasp	3	105.0	ISM
#4	54, m	Wasp	3	27.5	n.d.
#5	72, m	Wasp	3	29.2	ISM
#6	60, m	Wasp	3	20.9	n.d.
#7	62, m	Bee	3	23.1	CM
#8	43, f	Wasp	3	27.1	CM
#9	35, m	Bee	3	25.4	n.d.
#10	41, m	Bee	3	40.1	ISM
#11	46, m	Wasp	3	41.4	ISM
#12	53, m	Wasp	3	98.1	ISM
#13	49, m	Wasp	3	21.3	n.d.
#14	56, m	Wasp	3	25.4	n.d.
#15	51, f	Bee	3	28.6	ISM
#16	46, m	Wasp	3	25.2	CM
#17	69, m	Wasp	3	26.5	ISM
#18	74, m	Wasp	3	34.6	ISM
#19	53, m	Bee	3	23.0	n.d.
#20	43, m	Wasp	3	25.6	n.d.
#21	40, m	Wasp	2	28.6	n.d.
#22	43, m	Wasp	3	39.9	ISM
#23	54, f	Wasp	3	35.8	ISM
#24	40, m	Wasp	3	32.0	ISM
#25	29, m	Wasp	2	148.0	ISM
#26	36, m	Wasp	3	25.4	n.d.
#27	64, f	Wasp	3	32.0	ISM
#28	64, m	Wasp	2	20.6	n.d.
#29	31, m	Wasp	2	129.0	ISM

ISM, indolent systemic mastocytosis. CM, cutaneous mastocytosis. n.d., not determined yet.

### Diagnosis of mastocytosis

Diagnosis of indolent systemic mastocytosis could conclusively be achieved in 3 NSAID hypersensitive patients and in 15 hymenoptera venom allergic patients (Table 3, Table 4). Because of histologically proven skin involvement a preliminary diagnosis of mastocytosis in the skin according to current guidelines was made in 1 NSAID hypersensitive and in 3 hymenoptera venom allergic subjects with basal serum tryptase levels > 20 ng/mL [13]. Unfortunately, in several patients listed in Table 3 and Table 4 not all necessary clinical examinations for final classification of mastocytosis were performed [13,14]. These patients were labelled as "classification not determined yet".

### Statistical comparison between patients with NSAID hypersensitivity and hymenoptera venom allergy

Calculated *P* values are depicted in Table 2. As expected, age of patients, male–female ratio and severity of anaphylaxis were statistically significantly different, i.e. venom allergic patients are younger, are predominantly male, and suffered from more severe anaphylaxis. Compared with NSAID hypersensitive patients, basal serum tryptase levels were significantly higher in venom allergic subjects (*P* = .004).

## Discussion

In hymenoptera venom allergic subjects, raised baseline serum tryptase levels (obtained at least 1 week after resolution of the clinical signs) have been identified as a substantial risk factor for more severe anaphylaxis [5,16-18]. In accordance to these data, our group of hymenoptera venom allergic patients included a significant number with a history of severe anaphylactic reactions to hymenoptera stings and concomitantly elevated tryptase levels of > 20 ng/mL.

In contrast to immune-mediated allergy, the most important triggers of non-allergic drug-induced hypersensitivity are NSAID. The clinical symptoms of these reactions may closely mimic IgE-mediated mild to moderate anaphylaxis and mast cell degranulation has been demonstrated in IgE-mediated reactions as well as in non-allergic drug-induced hypersensitivity [3,19]. Together with a generally increased risk for anaphylaxis in patients with mastocytosis [3,4,11], and because of individual cases reporting about severe non-allergic drug hypersensitivity reactions in these patients, it has been concluded that mastocytosis patients may be predisposed for NSAID-induced reactions [6]. In our clinically well-defined cohort of patients with non-allergic NSAID hypersensitivity, elevated basal serum tryptase levels were found only in a small number of cases and elevated tryptase levels seemed to be not associated with more severe reactions. In contrast to hymenoptera venom allergic patients, baseline serum tryptase levels in our NSAID hypersensitive subjects did not differ from those published in a general adult population (mean 5.6 ng/mL) [20].

Up to date there have been a limited number of studies focusing on the association of mastocytosis and non-allergic drug hypersensitivity. In an Italian study, 7 out of 86 patients (8.1%) were identified with an elevated baseline serum tryptase of > 11.4 ng/mL, ranging from 13.2 to 26.9 ng/mL [17]. The incriminated drugs included acetylsalicylic acid (2x), β-lactam antibiotics (2x), chloramphenicol, radio contrast medium, and chlorhexidine, respectively. In this study, only one patient was diagnosed with indolent systemic mastocytosis. Another study identified in a cohort of 83 mastocytosis patients 16 (19.3%) individuals with a history of drug-induced anaphylaxis. The incriminated drugs in this study were β-lactam antibiotics (4x), fosfomycin, NSAID (4x), codeine, mepivacaine, and rocuronium and in 4 patients hypersensitivity reactions could not be attributed to a single identifiable drug [21]. In our cohort of 284 NSAID hypersensitive patients we identified 26 who showed basal serum tryptase levels of > 10 ng/mL. It was hypothesized that systemic hypersensitivity reactions in patients with mastocytosis tend to be more severe. We were not able to confirm this hypothesis because only 2 of 6 patients with serum tryptase levels of > 20 ng/mL had moderate (grade 2) anaphylaxis not requiring adrenaline, while the remaining 4 patients experienced merely symptoms of urticaria and angioedema.

The pathogenesis of NSAID-induced non-allergic hypersensitivity is believed to be related to cyclooxgenase 1 inhibition resulting in activation of mediator release from inflammatory cells in the skin and not to an IgE-mediated allergy [22]. Although the exact role of mast cells and histamine is still not clear, a predisposition for drug-induced non-allergic hypersensitivity has been postulated for mastocytosis patients. This resulted in recommendations to avoid NSAID in patients with confirmed mastocytosis. In cases of medical necessity, pre-treatment with H_1_-antihistamines and corticosteroids or graded challenges should be performed. However, our and the limited data in the literature failed to demonstrate a high risk for mast cell disease in patients with non-allergic NSAID hypersensitivity. Furthermore, the severity of hypersensitivity reactions was not increased in those patients with elevated basal tryptase levels with or without confirmed mastocytosis, suggesting that the recommendation to avoid NSAID may be not necessary.

While the association of mastocytosis and hymenoptera venom allergy has been confirmed in several studies, the role of drugs as triggers for anaphylaxis in patients with mast cell disease is still unclear. In our patients with non-allergic hypersensitivity reactions to NSAID basal tryptase levels appear not to be elevated and increased levels were not associated with a stronger severity of clinical reactions.

### Clinical implications

In patients suffering from NSAID hypersensitivity, determination of basal serum tryptase is unlikely to identify patients with elevated levels or mastocytosis and did not indicate increased reaction severity. This suggests that mastocytosis patients could be treated with this class of drugs without special precautions.

## Abbreviations

NSAID: Non-steroidal anti-inflammatory drug.

## Competing interests

The authors declare that they have no competing interests.

## Authors’ contribution

CS participated in the design of the study, collection and interpretation of data, and drafted the manuscript. KB participated in the design of the study and critically revised the manuscript. JH performed the statistical analysis, and helped to draft the manuscript. AT participated in the design of the study, collection and interpretation of data, and drafted the manuscript. All authors read and approved the final manuscript.
